# Characterization of an in vitro model to study CD4^+^ T cell metabolism in dairy cows

**DOI:** 10.3168/jdsc.2024-0565

**Published:** 2024-05-10

**Authors:** U. Arshad, M. Cid de la Paz, H.M. White, L.R. Cangiano

**Affiliations:** Department of Animal and Dairy Sciences, University of Wisconsin–Madison, Madison, WI 53706

## Abstract

Dairy cows are susceptible to several health disorders throughout their lactation. Objectives were to characterize an in vitro model to study bioenergetic measures in CD4^+^ T lymphocytes in dairy cows. Twenty-four healthy mid-lactation multiparous Holstein dairy cows were enrolled at a mean (±SD) of 234 ± 22 DIM. Cows were blocked according to DIM and blood was collected to isolate peripheral blood mononuclear cells followed by magnetic separation of CD4^+^ T lymphocytes using bovine-specific monoclonal antibodies. The isolated CD4^+^ T lymphocytes from each cow were split into 2 tubes and randomly assigned to incubate in an assay medium as control (CON) or with a combination of phorbol myristate acetate and ionomycin (PMA+IMY) to evaluate metabolic function under a resting and activated state. Mitochondrial and glycolytic functional kinetics were recorded in CD4^+^ T lymphocytes based on real-time measurement of oxygen consumption rate (OCR) and extracellular acidification rate (ECAR) under basal conditions and in response to complex V inhibitor (oligomycin), a protonophore uncoupler (BAM 15), and complex I and complex III inhibitors (rotenone and antimycin A). The mean (±SD) viability and purity of CD4^+^ T lymphocytes was 92.5 ± 2.9% and 95.2 ± 2.9%, respectively. The basal OCR in CD4^+^ T lymphocytes treated with PMA+IMY was greater than CON; nevertheless, the maximal respiration rate (CON = 58.0 vs. PMA+IMY = 47.3 ± 5.7 pmol/min) and sparing respiratory capacity rate (CON = 42.0 vs. PMA+IMY = 28.7 ± 4.2 pmol/min) were decreased in activated CD4^+^ T lymphocytes. The ECAR in CD4^+^ T lymphocytes increased progressively over time in PMA+IMY compared with CON, which indicated an increase in aerobic glycolysis in PMA+IMY compared with CON (CON = 46.9 vs. PMA+IMY = 86.4 ± 7.0 pmol/min). Activated CD4^+^ T lymphocytes exhibit a metabolic switch from oxidative phosphorylation to aerobic glycolysis, which may support rapid cell proliferation. The results observed in this experiment demonstrate the sensitivity of the technique to detect changes in metabolic function under different cellular conditions, providing a robust framework to study immuno-metabolism in dairy cattle.

## Highlights

• CD4^+^ T cells increased their metabolic activity after activation.
• Maximal mitochondrial respiration was reduced after a metabolic stress was induced.
• Activated CD4^+^ T cells switch from oxidative phosphorylation to aerobic glycolysis.
• This model can improve our understanding of how changes in metabolism affect immunity.

## Graphical Abstract

**Figure:**
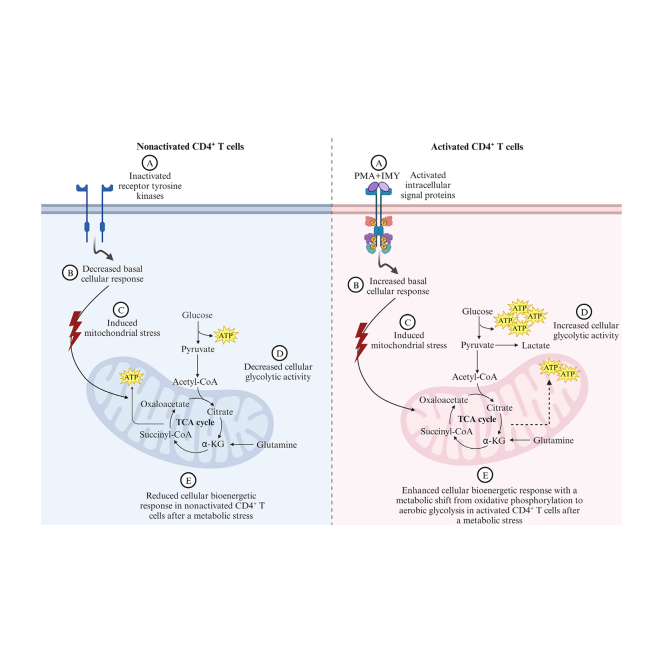
**Summary:** Study objectives were to characterize an in vitro model to study energy metabolism in CD4^+^ T cells in dairy cows. Twenty-four mid-lactation Holstein cows were enrolled, and peripheral blood mononuclear cells were isolated, followed by magnetic separation of CD4^+^ T lymphocytes. The CD4^+^ T lymphocytes either remained unstimulated (CON) or were treated with phorbol myristate acetate and ionomycin (PMA^+^IMY) to evaluate metabolic function under resting and activated states. Oxygen consumption and extracellular acidification rate were recorded in real time under basal conditions and in response to mitochondrial stress. The activation of CD4^+^ T lymphocytes induced increased basal respiration, decreased maximal respiration and sparing respiratory capacity, and increased aerobic glycolysis. Our findings reveal that activated CD4^+^ T lymphocytes exhibit a metabolic switch from oxidative phosphorylation to aerobic glycolysis. This model can become a useful tool to advance our understanding of how alterations in metabolism influence the overall health of dairy cows.

During early lactation, several physiological and metabolic changes occur to support lactation. However, some of these changes are linked to negative nutrient balance and immune suppression in dairy cows (Esposito et al., 2014), which contribute to an increased incidence of infectious diseases and metabolic disorders in this period (Chapinal et al., 2011). Developing strategies to improve health outcomes requires improving our understanding of the underlying molecular mechanisms that modulate the interplay between the immune system and its metabolic function. Using ex vivo models to assess the impact of different conditions or physiological stages on immune and metabolic function can provide a valuable means to understand the intricate mechanisms contributing to increased susceptibility to diseases during early lactation. The objective of this experiment was to characterize a model to study bioenergetic measures in vitro in CD4^+^ T lymphocytes in dairy cows using an extracellular flux analyzer. We hypothesized that the sensitivity of our extracellular flux analyzer model would capture the change in metabolic function experienced by activated CD4^+^ T lymphocytes subjected to mitochondrial stress. More specifically, we hypothesized that activated CD4^+^ T lymphocytes would experience a metabolic shift from oxidative phosphorylation to aerobic glycolysis.

The experiment was conducted at the University of Wisconsin–Madison, and all procedures with cows were approved by the Institutional Animal Care and Use Committee of the University of Wisconsin–Madison, protocol number A006420-A03. Twenty-four mid-lactation multiparous Holstein dairy cows at a mean (±SD) 234 ± 22 DIM were housed in freestalls at Emmons Blaine Dairy Cattle Center (Arlington, WI) for 2 wk. All cows were healthy and did not experience any health problems such as mastitis, pneumonia, lameness, or indigestion at least 40 d before enrolling in this study. Cows were allowed ad libitum access to feed and water during the experiment. Cows were blocked according to DIM, and each day, a set of 2 to 4 cows (1 to 2 blocks) were enrolled in the experiment. A total of 60 mL of blood was collected from the coccygeal vein of each cow before the morning milking at approximately 0630 h. Blood was collected in vacutainer K_2_EDTA tubes (Vacutainer, Becton Dickson, Franklin Lakes, NJ) and transferred to the laboratory at room temperature within 35 min after collection. Blood tubes were centrifuged at 1,200 × *g* for 15 min at room temperature and buffy coat was harvested and transferred into a 50-mL sterile tube. The buffy coat was diluted with 15 mL of PBS. Peripheral blood mononuclear cells (**PBMC**) were isolated by density gradient separation using Histopaque-1077 (product 10771, Sigma-Aldrich, St. Louis, MO) and washed twice in PBS. Red blood cells were removed by hypotonic lysis using double-distilled water. Purified PBMC were resuspended in PBS containing 0.5% BSA (Sigma-Aldrich). Peripheral blood mononuclear cells were counted using an automatic cell counter (Countess II FL, Invitrogen, Waltham, MA), and the viability of cells was assessed by diluting cell suspension with 0.08% (vol/vol) solution of trypan blue (product T8154, Sigma-Aldrich) in 1:1 dilution.

The CD4^+^ T lymphocytes were isolated using magnetic cell separation (Miltenyi Biotec, Bergisch Gladbach, Germany) according to the manufacturer's instructions. Briefly, aliquots of 1 × 10^7^ of PBMC were first incubated for 30 min at room temperature in the dark with 5 µL of anti-CD4 (product WS0562B-100, clone ILA11A, Kingfisher Biotech Inc., Saint Paul, MN) in 100 µL of PBS supplemented with 0.5% of BSA (PBS-BSA). Thereafter, PBMC were washed twice with PBS-BSA and incubated for an additional 15 min at 4°C by adding 20 µL of MACs IgG2a+b magnetic beads (product 130–047–202, Miltenyi Biotec) and 80 µL of PBS-BSA. Peripheral blood mononuclear cells were washed again with PBS supplemented with BSA, and the bead-CD4^+^ T lymphocytes complex was extracted with a LS magnetic-activated cell sorting column (product 130–042–401, Miltenyi Biotec) according to the manufacturer's instructions. During the first incubation of PBMC with the ILA11A antibody, PBMC were also incubated with 10 µL of anti-CD4 conjugated to Alexa Fluor 700 (product MCA1038A700, Bio-Rad, Hercules, CA) to determine the purity of the isolated CD4^+^ T lymphocytes population. The purity of CD4^+^ T lymphocytes was assessed by flow cytometry (Attune NxT Flow Cytometer, Thermo Fisher Scientific, Waltham, MA). The viability of sorted CD4^+^ T lymphocytes was assessed using 0.08% trypan blue solution in an automatic cell counter (Countess II FL, Invitrogen). The CD4^+^ T lymphocytes were counted to prepare 2 aliquots containing 2.4 × 10^6^ cells in each aliquot for downstream Seahorse analysis.

The Seahorse analysis was performed to evaluate bioenergetics measures in CD4^+^ T lymphocytes using an XFp T cell metabolic profiling kit (product 103771–100, Agilent Technologies Inc., Santa Clara, CA). The day before analysis, Agilent Seahorse XFp Extracellular Flux Cartridge (product 103022–100, Agilent Technologies Inc.) was hydrated according to the manufacturer's instructions and placed in a non-CO_2_ incubator overnight at 37°C. Additionally, approximately 5 mL of XF Calibrant medium (product 103022–100, Agilent Technologies Inc.) for the hydration of each utility plate of cartridge and XFp PDL miniplate (product 103722–100, Agilent Technologies Inc.) were placed in a non-CO_2_ incubator overnight at 37°C. On the day of analysis, the standard Seahorse XF RPMI assay medium was prepared by supplementing 19.4 mL of Seahorse XF RPMI medium (product 103576–100, Agilent Technologies Inc.) with 0.2 mL each of glucose (product 103577–100, XF 1.0 *M* Glucose Solution, Agilent Technologies Inc.), pyruvate (product 103578–100, XF 100 m*M* Pyruvate Solution, Agilent Technologies Inc.), and glutamine (product 103579–100, XF 200 m*M* Glutamine Solution, Agilent Technologies Inc.) solutions to achieve final concentrations of 10, 1, and 2 m*M*, respectively, and stored at 37°C throughout the assay.

The isolated CD4^+^ T lymphocytes from each cow were split into 2 tubes and randomly assigned to incubate with either 250 µL of XF RPMI assay medium as control (**CON**) or treated with a combination of 140 µL of Seahorse XF RPMI assay medium, 100 µL of phorbol 12-myristate 13-acetate (product J63916-M, Thermo Fisher Scientific) at 20 ng/mL, and 10 µL of ionomycin (product I24222, Invitrogen) at 1 µg/mL (**PMA+IMY**) for 2 h at 37°C. The rationale for incubating CD4+ T lymphocytes with a combination of PMA+IMY was to induce immune activation and promote high metabolic activity in immune cells. This methodology stemmed from the observed increase of cytokine production by immune cells when exposed to this combination (Ai et al., 2013). After 2 h incubation of CD4^+^ T lymphocytes with treatments, 50 µL of CD4^+^ T lymphocytes suspension from each tube was added in triplicate to have 4.0 × 10^5^ live cells density per well in the XFp PDL miniplate (Eder et al., 2020). Thereafter, 50 µL of Seahorse XF RPMI assay medium was added to each well, and the moats around each well were filled with 200 µL of warm sterile water. The XFp PDL miniplate was centrifuged at 200 × *g* for 1 min at room temperature to allow cells to attach to the bottom of the wells, and an additional 100 µL of warm XF RPMI assay medium was added in each well. Thereafter, the XFp PDL miniplate was incubated at 37°C in a non-CO_2_ incubator for 60 min before performing the T cell persistence assay. Meanwhile, on the day of the analysis, the XFp Extracellular Flux Cartridge was removed from the incubator and rehydrated with prewarmed XF Calibrant media according to the manufacturer's instructions. The cartridge was then placed back into the non-CO_2_ incubator at 37°C for at least 60 min before adding the port injection substrates. The port injection substrates in XFp T cell metabolic profiling kit included complex V inhibitor (oligomycin), a protonophore uncoupler (BAM 15), and complex I and complex III inhibitors (rotenone and antimycin A). Each reagent was resuspended with 0.5 mL of warm XF RPMI assay medium to achieve final concentrations of 13.5 µ*M* oligomycin, 25 µ*M* BAM 15, and 5.5 µ*M* rotenone and antimycin A. Twenty-five microliters of oligomycin, BAM 15, and rotenone and antimycin A were added in ports A, B, and C, respectively, for each well of XFp PDL miniplate. The rationale to add these reagents in injection ports was to induce mitochondrial stress in CD4^+^ T lymphocytes and measure the bioenergetic changes in those cells. After injection substrates were added to each port, the cartridge was placed in Seahorse for calibration. Once the cartridge was calibrated, the XFp PDL miniplate was inserted into the Seahorse machine for analysis to perform the T cell persistence assay. The assay was conducted in triplicate, and 2 wells within each PDL miniplate were designated for quality control (medium without cells). Data were exported and quality of data examined using the raw Excel outputs and Seahorse Wave Pro Software (version 10.1.0, Agilent Technologies Inc., 2021). During this assay, mitochondrial function kinetics were recorded in real time, measuring oxygen consumption rate (**OCR**; pmol/min) as a marker for oxidative phosphorylation, whereas cytoplasmic pH changes were monitored in real time measuring the extracellular acidification rate (**ECAR**; mpH/min) as an indicator of cellular glycolysis. Additionally, the proton efflux rate (**PER**; pmolH^+^/min) was measured, which represents a quantitative assessment of extracellular acidification. The measures of bioenergetics were calculated using Seahorse Analytics (version 1.0.0–570, Agilent Technologies Inc., 2023) and included mitochondrial ATP production rate (pmol/min), glycolytic ATP production rate (pmol/min), total ATP production rate (pmol/min), basal respiration rate (pmol/min), maximal respiration rate (pmol/min), and sparing respiratory capacity (**SRC**; pmol/min) rate. The bioenergetic measures for each plate were averaged across each triplicate.

The experiment followed a randomized complete block design. Cows were ranked by DIM, from the smallest to the largest value, and then every 2 cows were assigned to a block. Within each block, CD4^+^ T lymphocytes were isolated from each cow and split into 2 tubes, which were assigned randomly to CON or PMA+IMY treatments; therefore, the tube was the experimental unit in this experiment. Normality of residuals and homogeneity of variance were examined for each continuous dependent variable analyzed after fitting the statistical model. Some responses (OCR, ECAR, and PER) violated the assumptions of normality and were subjected to power transformation according to the Box-Cox procedure (Box and Cox, 1964) using PROC TRANSREG in SAS (SAS/STAT, SAS Institute Inc.); however, the interpretation of data did not change after transformation. Therefore, analysis of untransformed data was used, and the LSM and respective SEM were presented on the original scale to avoid back transformation of SEM. Data were analyzed by mixed-effects models using the MIXED procedure of SAS (ver. 9.4, SAS/STAT, SAS Institute Inc., Cary, NC). The statistical models included the fixed effects of treatment (CON vs. PMA+IMY), time (min) of measurement, the interaction between treatment and time, and the random effects of block and tube nested within treatment. In all statistical models with repeated measures, the REPEATED statement was used for dependent variables measured over time. The tube nested within treatment was the error term for testing the effect of treatment. The response variables had equal spacing between measurements; therefore, the autoregressive 1 was the most selected covariance structure. When an interaction between treatment and time resulted in *P* < 0.10, then treatment means at different time points were partitioned using the SLICE command of SAS (SAS/STAT, SAS Institute Inc.). The Kenward-Roger method was used to approximate the denominator degrees of freedom to compute the *F* tests. Evidence of statistical significance against the null hypothesis was considered at *P* ≤ 0.05, and a tendency was considered at 0.05 < *P* ≤ 0.10.

All 24 cows contributed data to this experiment. The mean (±SD) viability of PBMC and CD4^+^ T lymphocytes were 92.1 ± 2.8% and 92.5 ± 2.9%, respectively. The mean (±SD) purity of CD4^+^ T lymphocytes after isolation from PBMC was 95.2 ± 2.5%, and dot plots from a representative cow are presented in Figure 1A–D. The OCR was affected by the interaction (*P* = 0.002) between treatment and time (Figure 2A). The interaction was caused by the greater increase in basal OCR in CD4^+^ T lymphocytes treated with PMA+IMY compared with CON; nevertheless, the OCR in CD4^+^ T lymphocytes after adding an uncoupler (i.e., BAM 15) was increased in CON compared with PMA+IMY (Figure 2A). The mitochondrial ATP production rate tended (*P* = 0.06) to be reduced in PMA+IMY compared with CON (CON = 100.3 vs. PMA+IMY = 115.6 ± 7.6 pmol/min; Figure 2B). The ECAR in CD4^+^ T lymphocytes increased (*P* < 0.0001) progressively over time in PMA+IMY compared with CON (Figure 2C), which indicated an increase (*P* < 0.0001) in aerobic glycolysis in PMA+IMY compared with CON (CON = 46.9 vs. PMA+IMY = 86.4 ± 7.0 pmol/min; Figure 2D). Similarly, the PER in CD4^+^ T lymphocytes increased (*P* < 0.0001) progressively over time in PMA+IMY compared with CON (Figure 2E). The total ATP production rate was greater (*P* = 0.001) in PMA+IMY compared with CON (CON = 143.4 vs. PMA+IMY = 193.4 ± 17.5 pmol/min; Figure 2F). Based on the OCR changes before and after the induction of mitochondrial stress, the basal respiration rate was greater (*P* = 0.05) in CD4^+^ T lymphocytes treated with PMA+IMY compared with CON (Table 1). The maximal respiration rate tended (*P* = 0.07) to decrease in CD4^+^ T lymphocytes treated with PMA+IMY compared with CON (Table 1). Since the ability of CD4^+^ T lymphocytes to consume oxygen after mitochondrial stress was reduced, the SRC rate was also reduced in PMA+IMY compared with CON (Table 1).

**Figure 1:**
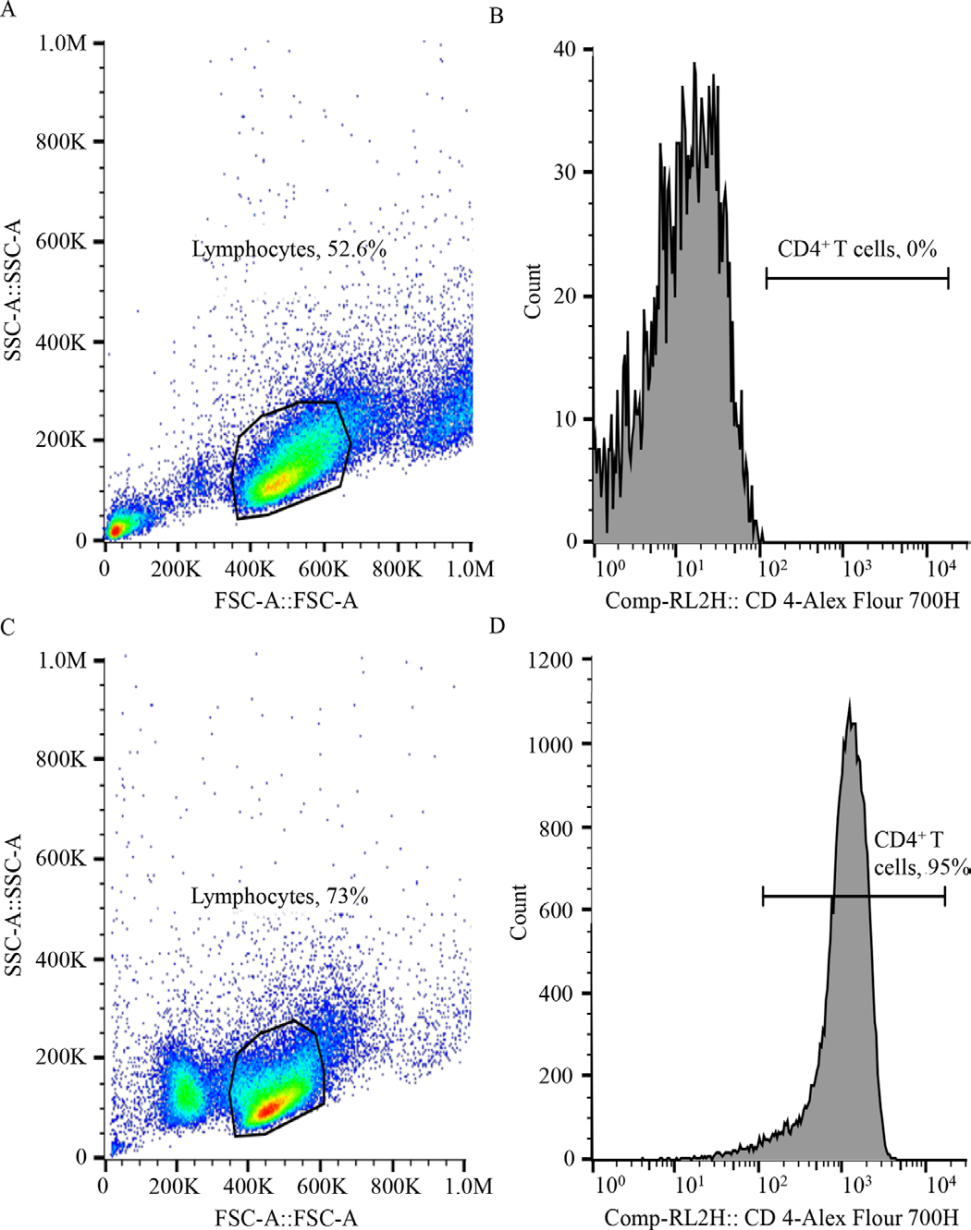
Gating strategy to determine purity of CD4^+^ T lymphocytes after their isolation by magnetic cell sorting from PBMC. Data were collected with an Attune NxT Flow Cytometer (Thermo Fisher Scientific). Lymphocytes were identified using forward scatter (FSC) and side scatter (SSC) parameters followed by expression of the coreceptor CD4^+^. Panels A and B indicate that PBMC were not incubated with anti-CD4^+^ antibody and served as a control group. Panels C and D represent the CD4^+^ enriched population from PBMC that were incubated with an anti-CD4^+^ antibody conjugated with Alexa Fluor 700 (Bio-Rad). The CD4^+^ T lymphocytes were identified by gating the lymphocyte population (panels A and C), followed by CD4^+^ T lymphocyte expression in channel RL2 (panels B and D). The control group contained 0% of CD4^+^ T lymphocytes (panel B), and the positive group represented 95% of CD4^+^ T lymphocytes (panel D).

**Figure 2:**
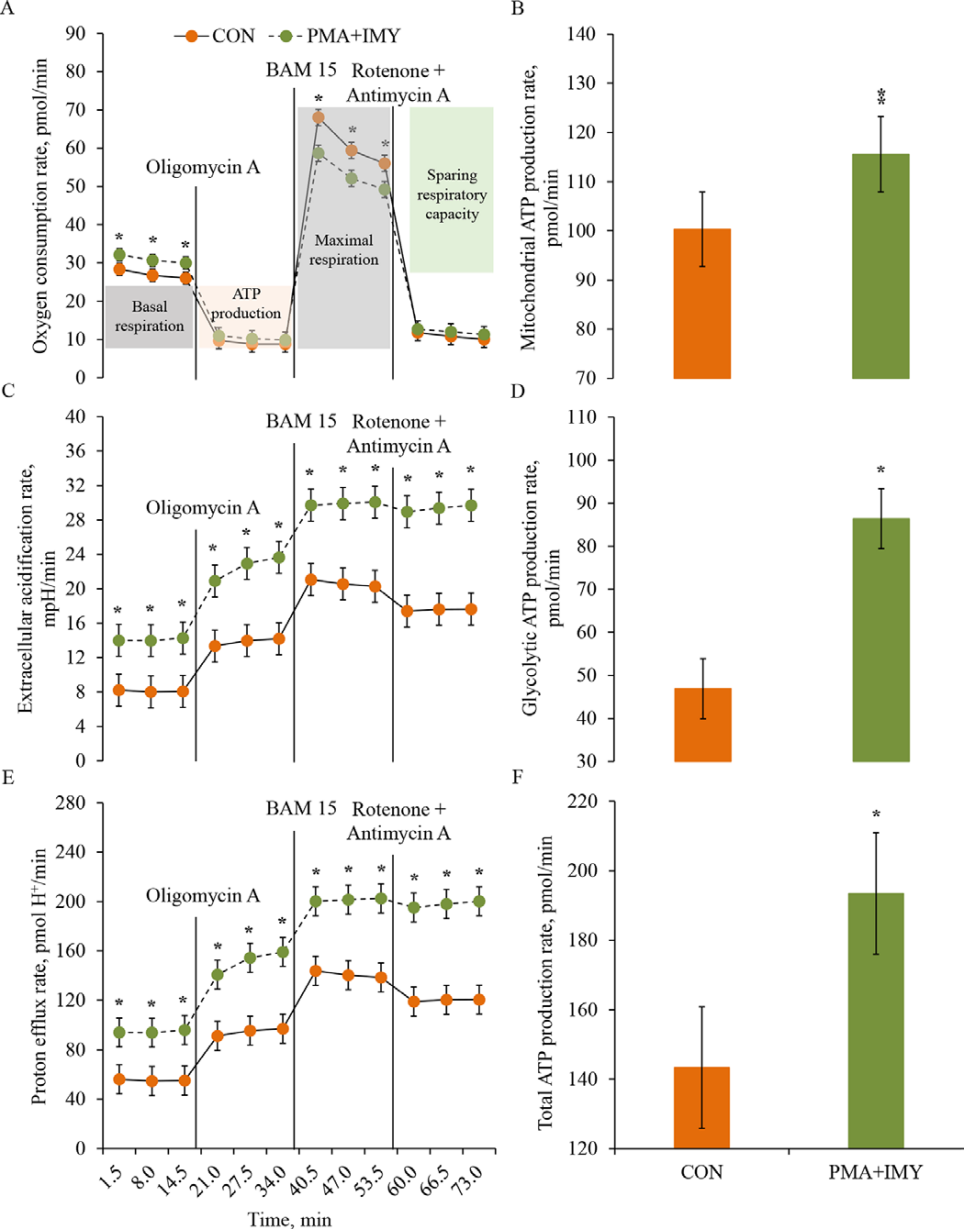
Oxygen consumption rate (OCR; A), mitochondrial ATP production rate (B), extracellular acidification rate (ECAR; C), glycolytic ATP production rate (D), proton efflux rate (PER; E), and total ATP production rate (F) in peripheral blood CD4^+^ T lymphocytes from mid-lactation dairy cows. The CD4^+^ T lymphocytes either remained unstimulated (CON) or were activated using a combination of phorbol myristate acetate and ionomycin (PMA+IMY) to evaluate measures of bioenergetics. Mitochondrial and glycolytic functional kinetics were recorded in real-time measuring OCR and ECAR, respectively, under basal conditions and in response to complex V inhibitor (oligomycin), a protonophore uncoupler (BAM 15), and complex I and complex III inhibitors (rotenone and antimycin A). Bioenergetic measures included basal respiration, maximal respiration, ATP production, and SRC in CD4^+^ T lymphocytes. Panel A: effects of treatment (*P* = 0.87), time (*P* < 0.0001), and interaction between treatment and time (*P* = 0.002). Panel B: effect of treatment (*P* = 0.06). Panel C: effects of treatment (*P* < 0.0001), time (*P* < 0.0001), and interaction between treatment and time (*P* < 0.0001). Panel D: effect of treatment (*P* < 0.0001). Panel E: effects of treatment (*P* < 0.0001), time (*P* < 0.0001), and interaction between treatment and time (*P* < 0.0001). Panel F: effect of treatment (*P* = 0.001). Symbols indicate level of significance (*P ≤ 0.05; **0.05 < *P* ≤ 0.10). Error bars depict SEM.

**Table 1:** Effect of treatment on bioenergetic measures in peripheral blood CD4^+^ T lymphocytes from mid-lactation dairy cows

Item	Treatment^1^		SEM	*P-*value
	CON	PMA+IMY		
Basal respiration rate, pmol/min	16.0	18.7	1.8	0.05
Maximal respiration rate, pmol/min	58.0	47.3	5.7	0.07
SRC rate, pmol/min	42.0	28.7	4.2	0.001

1 The CD4^+^ T lymphocytes either remained unstimulated (CON) or were activated using a combination of phorbol myristate acetate and ionomycin (PMA+IMY) to evaluate measures of bioenergetics.

Incubation of CD4^+^ T lymphocytes with PMA+IMY increased basal OCR, coinciding with increased basal mitochondrial and glycolytic ATP production rates, suggesting an increased basal metabolic activity. It is noteworthy to mention that CD4^+^ T lymphocytes were isolated from PBMC of mid-lactation cows, and previously it had been shown that immune responses are greatly dependent on stage of lactation in dairy cows (Shafer-Weaver et al., 1999). The rationale for conducting this study with mid-lactation cows was to mitigate the confounding effect of the higher disease incidence typically observed in early lactation while establishing this methodological approach. Eder et al. (2020) conducted an experiment to study metabolic reprogramming of activated and nonactivated CD4^+^ T lymphocytes in early, mid, and late lactation and dry period in healthy Holstein cows. The authors showed that mitochondrial function did not differ between stimulated and nonstimulated CD4^+^ T lymphocytes. In contrast, glycolytic rate and an increased ability to use glycolysis at a higher capacity were greater in stimulated CD4^+^ T cells during late lactation and dry period compared with stimulated CD4^+^ T cells from cows in early and mid lactation (Eder et al., 2020). Cows in our study represent late lactation; thus, our results are in close agreement with the findings of Eder et al. (2020) indicating that glycolytic pathways were upregulated in activated CD4^+^ T lymphocytes, and this metabolic rewiring in T lymphocytes is a conserved mechanism orchestrated to meet the specific bioenergetic and biosynthetic demands of activated immune system (Palsson-McDermott and O'Neill, 2013). In the present experiment, activated CD4^+^ T lymphocytes experienced a metabolic switch from oxidative phosphorylation to aerobic glycolysis. This metabolic switch, known as the “Warburg effect,” provides readily available energy and substrates for accelerated growth and proliferation (Warburg, 1956) and ensures an adequate fuel supply to activated immune cells (Vander Heiden et al., 2009). In this experiment, the main contribution in total basal ATP production rate in activated CD4^+^ T lymphocytes was through aerobic glycolysis. Although aerobic glycolysis is less efficient than oxidative phosphorylation in the amount of ATP produced per mole of glucose, aerobic glycolysis can generate ATP at a much faster rate (Locasale and Cantley, 2011). Additionally, the glycolytic reprogramming facilitates the production of metabolic intermediates important for cell growth and proliferation and provides a way to maintain redox balance (NAD^+^/NADH) in the cell (Vander Heiden et al., 2009; Anastasiou et al., 2011).

Across life cycle and physiological stages, having ex vivo models and methods that can be employed to track the impact of diverse conditions or challenges on immune and metabolic functions might offer alternative approaches to unravel the mechanism of immune function, and energetics in ruminants. The application of this model would be useful to study individual animal variance in immune cell metabolism during steady state, in response to challenges, during disease mechanisms, with nutritional interventions, or during different physiological states. For example, cows in the initial weeks of lactation have increased susceptibility to infectious diseases (Abuelo et al., 2023; Bradford and Contreras, 2024), which is often attributed to a period of compromised immunity, likely resulting from metabolic adaptations to negative energy balance (Ospina et al., 2010). Understanding the role of immune response, nutrient use, and immunometabolism during this period could provide valuable insight into how to improve management and reduce the incidence of metabolic and infectious disorders in the peripartum period. In contrast, these tools could also be valuable to determine differences across cows influenced by genetic selection or across a range of phenotypes (i.e., feed efficiencies, or milk production levels) that may all exhibit immunocompetence but have nuanced differences in immune responsiveness or nutrient use. Across these ranges of life cycle and physiological states, employing methods such as those described herein could be advantageous for investigating the individual variability among animals concerning immune cell metabolism.
